# Target detection and classification via EfficientDet and CNN over unmanned aerial vehicles

**DOI:** 10.3389/fnbot.2024.1448538

**Published:** 2024-08-30

**Authors:** Muhammad Ovais Yusuf, Muhammad Hanzla, Naif Al Mudawi, Touseef Sadiq, Bayan Alabdullah, Hameedur Rahman, Asaad Algarni

**Affiliations:** ^1^Faculty of Computing ad AI, Air University, Islamabad, Pakistan; ^2^Department of Computer Science, College of Computer Science and Information System, Najran University, Najran, Saudi Arabia; ^3^Centre for Artificial Intelligence Research, Department of Information and Communication Technology, University of Agder, Grimstad, Norway; ^4^Department of Information Systems, College of Computer and Information Sciences, Princess Nourah bint Abdulrahman University, Riyadh, Saudi Arabia; ^5^Department of Computer Sciences, Faculty of Computing and Information Technology, Northern Border University, Rafha, Saudi Arabia

**Keywords:** deep learning, unmanned aerial vehicles, remote sensing, dynamic environments, path planning, multi-objects recognition deep learning, multi-objects recognition

## Abstract

**Introduction:**

Advanced traffic monitoring systems face significant challenges in vehicle detection and classification. Conventional methods often require substantial computational resources and struggle to adapt to diverse data collection methods.

**Methods:**

This research introduces an innovative technique for classifying and recognizing vehicles in aerial image sequences. The proposed model encompasses several phases, starting with image enhancement through noise reduction and Contrast Limited Adaptive Histogram Equalization (CLAHE). Following this, contour-based segmentation and Fuzzy C-means segmentation (FCM) are applied to identify foreground objects. Vehicle detection and identification are performed using EfficientDet. For feature extraction, Accelerated KAZE (AKAZE), Oriented FAST and Rotated BRIEF (ORB), and Scale Invariant Feature Transform (SIFT) are utilized. Object classification is achieved through a Convolutional Neural Network (CNN) and ResNet Residual Network.

**Results:**

The proposed method demonstrates improved performance over previous approaches. Experiments on datasets including Vehicle Aerial Imagery from a Drone (VAID) and Unmanned Aerial Vehicle Intruder Dataset (UAVID) reveal that the model achieves an accuracy of 96.6% on UAVID and 97% on VAID.

**Discussion:**

The results indicate that the proposed model significantly enhances vehicle detection and classification in aerial images, surpassing existing methods and offering notable improvements for traffic monitoring systems.

## 1 Introduction

Experts are searching for methods to locate and categorize vehicles in the dynamic field of intelligent traffic management systems to improve the accuracy and usefulness of surveillance technologies (Pethiyagoda et al., 2023; Ren et al., 2024; Sun et al., 2020). This work examines the complex issues associated with aerial imaging, where precise vehicle detection is necessary, among other things, for parking management systems and crowded area detection. Previously, motion estimates inside picture pixels (Kumar, 2023; Sun et al., 2023) were used for vehicle identification in remote sensing data. This was an inefficient method that detected activity in areas that were not meant for detection. Recent developments in techniques such as object segmentation, silhouette extraction, and feature extraction combined with classification have improved the capacity to recognize objects in aerial images (Xiao et al., 2023a; Xiao et al., 2023b; Wang J. et al., 2024; Wang Y. et al., 2024). Aerial photographs perform various functions, such as carrying out emergency relief operations, maintenance of crop fields, and detecting forested areas due to their view completeness (Qu et al., 2023; Ding et al., 2021). Here, the system is based on the work of satellites; in the process of determining and sometimes providing time-sensitive transit ID of vehicles through the organization of the aerial video to reveal individual frames necessary for effective analysis. A preprocessing procedure that uses methods which are brightness enhancement, noise-reduction, normalization, and Contrast-Limited Adaptive Histogram Equalization (CLAHE) is helpful in simplifying the image and to spot the vehicles and thus the speed of the cars easily. Cars in complex landscapes are picked out using Fuzzy C Mean segmentation method. Here is the summary in bullet form: Aerial images are reliable for various tasks just because of their exceptional view. An approach was employed to transform moving aerial videos into image frames which are subjected to vehicle identification and classification (Ahmed and Jalal, 2024a; Ahmed and Jalal, 2024b). Processing steps are brightness enhancement, noise reduction, normalization, and Contrast Limited Adaptive Histogram Equalization (CLAHE) so that images become easily identifiable. Fuzzy C Mean segmentation technique that is used to find vehicles in complicated backgrounds (Yan, 2022; Chen et al., 2022a; Chen et al., 2022b; Li et al., 2023). YOLOv4, a brand newer model that is capable of precisely delineating objects bearing consideration to the fact that they are of small sizes, is used in the identifying stage. SIFT and ORB are the two feature extraction techniques that are employed to increase the accuracy of the classifier (Shang, 2023; Naseer et al., 2024). The algorithms provided by these networks benefit in size, position, and rotation invariance, as they can precisely identify vehicles in aerial view. As for a Convolutional Neural Network (CNN) classifier being deployed for vehicle classification, the new model will provide better classification outcomes and performance is much higher compared to the competition. There have been many investigations over the past couple of years concerning the recognition and classification of vehicles in aerial image sequences (Nosheen et al., 2024). Due to the complexity of the problem, it has been looked at in different ways by using various CNN network architectures and object detection algorithms. One of the most well-known object identification systems, Faster R-CNN (Region-based convolutional neural networks), was published by Ren et al. (2017) and Khan et al. (2024). After object extraction using RPN, Faster R-CNN performs box regression and classification within a rectangular region. Over the year, it has shown remarkable results in tracking and surveillance in a variety of object detection scenarios. Better performance was obtained by the Faster R-CNN model when tested on the task of identifying automobiles in aerial data. However, the computational complexity of Faster R-CNN can limit its real-time applications (Wang S. et al., 2022; Liu et al., 2023).

Object recognition by another major method is also commonly used. It is known as SSD (Single Shot MultiBox Detector), developed with Near-Real Time Object Detection Application by Liu et al. (2016), Wang J. et al. (2024), and Wang Y. et al. (2024). SSD is a model that stands for “Scale Spoofing Detection” and it is famous for high grade accuracy and reliability in detecting various scaled objects. Through SSD, several scholars have succeeded in consuming audio speed information while still preserving the optimum precision in vehicle identification. SSD handles either vehicle size differences, or perspective shift that may appear in distance aerial images using anchor boxes and multi-scale feature maps. Interest in YOLO (You Only Look Once) algorithms have they are being as high as it is today due to the real-time efficiency of the object detection algorithms they use. YOLO is grid-based classifier that produces educations and boundaries classes using the model’s grid (Zhou et al., 2022; Ansar et al., 2022).

YOLOv4, the latest version of the YOLO method has great detecting resolution that is faster than others. Studies have been carried out to check pictures of cars in an atmosphere taken using the YOLOv4 and the data shows that the algorithm has a high detection accuracy in real-time performance. Yusuf et al. (2024a), Yusuf et al. (2024b), and Alazeb et al. (2024) introduced the ResNet (Residual Network), another well-known CNN architecture that addresses the vanishing gradient issue and allows the training of extremely deep networks. ResNet has been applied to vehicle classification in aerial data in several studies, with better results than conventional CNN designs. Deep networks can be built utilizing ResNet’s skip connections and residual blocks without experiencing performance deterioration (Chen et al., 2023; Almujally et al., 2024).

Using state-of-the-art methods and procedures, this comprehensive approach forms the basis of our system’s main contributions:

- To optimize both model simplicity and image quality, our approach incorporates CLAHE and noise reduction techniques in the pre-processing stage.
- EfficientDet improves the detection objects capacity especially in segmented photos. We solve the problem of recognizing objects of different sizes with the application of improving vehicle detection level.
- Our model provides for precise identification of vehicles in aerial pictures which is made possible by combining scale as well as rotation-invariant features, like vectors of 2D and fast local features using the SIFT and ORB approaches. A Convolutional Neural Network (CNN) classifier system is how the model increases its efficiency in the classification cycle, so the increased accuracy consequently arises. It emphasizes the role of both valuable and crucial tensors to create a more effective aerial vehicle detection and classification model.

The article’s structure is as follows: Section II covers system architecture which saves the system from exposure to physical attacks. Section III takes the system to the next level with a rigorous experiment phase analysis of how the prototype works. Next, Section IV emerges summarizing the system’s discoveries and indicating the directions of further transformations.

## 2 Literature review

Recently, various studies on vehicle detection and tracking have been carried out. The most recent techniques and methods used in these systems will be covered in more depth in this section. For better comprehension, we separate the literature into two main streams: vehicle tracking and detection.

### 2.1 Vehicle detection algorithms

Li et al. (2021) used spatial pyramid pooling (SPP) in conjunction with convolutional neural networks (CNN) to achieve high accuracy vehicle identification in complicated environments. The SPP module collected context information at many scales, which allowed the model to account for changes in vehicle sizes even though CNN was successful in extracting hierarchical features from the input pictures. The combination of these methods enhanced detection performance, especially in difficult situations when there were occlusions and thick backgrounds. A deep learning technique based on the Faster R-CNN architecture was reported by Zhang et al. (2017). An RPN was used to propose areas, while a CNN was utilized to extract features. Together, these parts gave the system the capacity to precisely anticipate bounding boxes, which let it identify and locate cars in real time. The efficacy and efficiency of the algorithm in addressing vehicle identification tasks were evaluated via the use of RPN and deep learning. A comprehensive process chain for exploiting synthetic data in vehicle detection is evaluated by Krump and Stütz (2023). Learning models with various configurations of training data and assessing the resultant detection performance are part of the research. The process of creating synthetic training data includes these assessments. The authors look at the possibility of improving detection performance in the last phase. A real-time vehicle detection technique based on deep learning was reported by Javed Mehedi Shamrat et al. (2022). The strategy was to develop a lightweight CNN architecture that would enable efficient vehicle detection on low-resource devices. By making the model and its parameters simpler, the approach achieved real-time performance without reducing detection accuracy. This approach worked well for applications requiring embedded systems or low-power devices, when computational resources were limited. Ma and Xue (2024) presented a novel method for vehicle detection that utilizes a deformable component model (DPM) in conjunction with a convolutional neural network (CNN). The DPM modeled the spatial interaction between different components to capture the structural attributes of vehicles; the CNN recovered discriminative information for accurate identification. Together, these two techniques improved vehicle recognition resilience and accuracy, especially under challenging circumstances with obstacles and shifting perspectives. A two-stage framework for vehicle detection was suggested by Kong et al. (2023) that makes efficient use of previous attribution information of cars in aerial images. The approach solves the scale variation issue by making use of convolutional layers with various receptive fields. The framework, which has been verified on difficult datasets like AI-TOD and view, shows promise in vehicle recognition. It consists of a Parallel RPN, Density-assigner, and Scale-NMS. Zhu et al. (2022) modifies the image segmentation technique to unify the vehicle size in the input image, simplifying the model structure and enhancing the detection speed. It also suggests a single-scale rapid convolutional neural network (SSRD-Net) and designs a global relational (GR) block to enhance the fusion of local and global features. A vehicle identification method based on deep learning and attention processes was developed by Zhang et al. (2021). The proposed strategy for highlighting important components and suppressing irrelevant ones includes the self-attention mechanism. By identifying the distinctive features of cars, even in crowded environments where they can be obscured by other clutter, the system increased the accuracy of detection. The result of the attention process, which increased the discriminative capability of the model, was better detection performance. Yilmaz et al. (2018) suggested maximizing the success rate of a trained detector by examining several factors, including the convolutional neural network’s architecture, the setup of its training choices, and the Faster R-CNN object detector’s training and assessment. The research shows that by effectively recognizing automobiles in real-time settings, the most recent approaches in vehicle detection systems specifically, R-CNN and Faster R-CNN deep learning methods significantly improve traffic management.

### 2.2 Vehicle tracking algorithms

Vehicle tracking algorithms have taken the stage in automated traffic monitoring research as the rising need for effective surveillance systems drives. Important research and approaches in the realm of literature are thoroughly analyzed in this part. Focusing on several sensors and algorithms, the authors (Wang et al., 2022) investigated their possibilities and uses in intelligent automobile systems using a fresh method. The article also addresses possible future directions and difficulties and looks at ways to identify vehicles in bad weather. Wood et al. (1985) demonstrated how well the Kalman filter, a widely used real-time tracker in computer vision kept vehicle tracks across numerous picture frames. This highlights how very important the Kalman filter is for applications in vehicle tracking. Long-term tracking using SURF-based optical flow was first presented by the authors (Li et al., 2012). This technique combines online visual learning and computes warp matrices around SURF critical sites. In complicated real-world situations, this method shows much better tracking performance than conventional optical flow systems (Qiu et al., 2020) integrated the YOLO object detector with the Deep SORT algorithm, yielding a robust solution for real-time vehicle surveillance that delivers unparalleled precision and efficiency. Vermaak et al. (2005) tackled the difficulties of multi-object tracking by introducing a method that relies on the Joint Probabilistic Data Association Filter (JPDAF). This technique can handle occlusions and maintain correct track connections in tough settings. Meanwhile, Guo et al. (2022) created an improved vehicle tracking system that blends deep learning-based object detection techniques with the Hungarian algorithm for track association. This system displays great performance, even under hectic traffic circumstances. In addition (Tayara et al., 2017) proposed a unique approach to enhance vehicle tracking in aerial pictures. They merged object-level and trajectory-level properties using convolutional neural networks (CNNs) and recurrent neural networks (RNNs). This resulted in better precision and reliability in tracking. This essential research illustrates the amazing efficacy and flexibility of different algorithms used for monitoring automobiles. These algorithms include breakthroughs in graph-based approaches, Kalman filtering, feature extraction techniques, deep learning, and association algorithms. Therefore, these studies are defining the direction of current research on vehicle tracking, with the objective of building more efficient and reliable surveillance systems that may be utilized in a broad variety of real-world applications.

## 3 Materials and methods

### 3.1 System methodology

**Figure 1:**
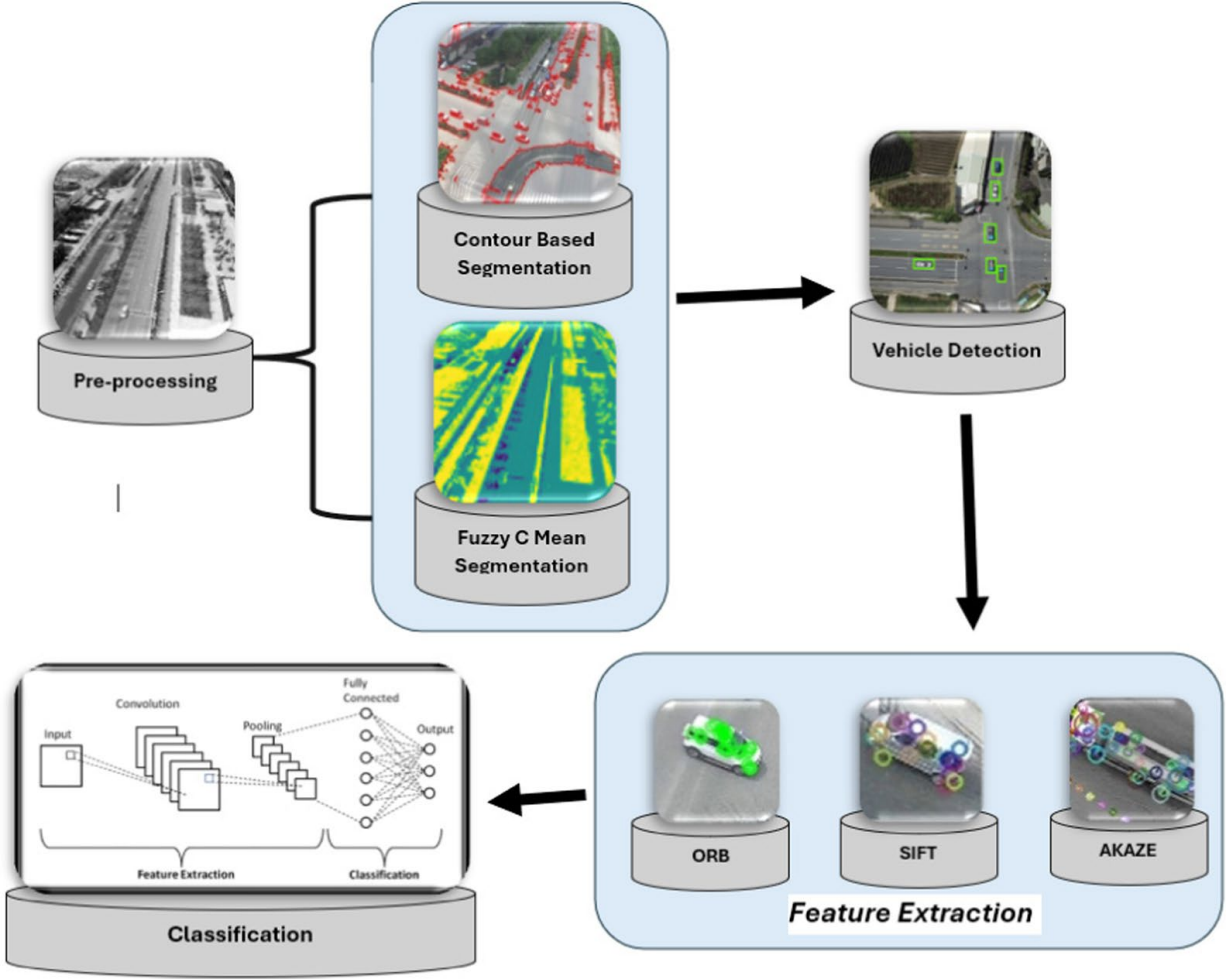
The architecture of the proposed system.

The recommended design is based on a sophisticated item recognition and vehicle categorization approach applied for the region under evaluation. The procedure begins with the conversion of aerial images into individual frames. Before the detection phase, these frames go through important pre-processing procedures. i.e., Noise removal and CLAHE is used to improve image intensity dynamically, hence maximizing the visual input for subsequent detection algorithms. Following pre-processing, Fuzzy C Mean (FCM) segmentation is used to successfully discriminate foreground and background objects in the filtered images, resulting in a refined input for subsequent analysis. EfficientDet powers the detection phase. After the detection SIFT and ORB approaches are used to extract features from detected vehicles. This feature vector, which contains comprehensive details about the observed vehicles, serves as input for the CNN classifier. Figure 1 depicts the overall architecture of the suggested approach. The proposed system’s architecture is visually represented in Figure 1.

### 3.2 Pre-processing

Through the disseminating of pixel intensity levels, histogram equalization improves contrast in an image, improving features and streamlining research (Huang et al., 2023; Zhang et al., 2019). Applying the approach to color images, one can equalize the luminance channel in a color structure that differentiates between luminance and color information, such the YCrCb color space, guaranteeing improved image quality and feature visibility (Gong et al., 2018; Pervaiz et al., 2023). The process involves creating the cumulative distribution function (CDF) for transforming the pixel values, calculating the histogram of the original image, and mapping every pixel to a new intensity value based on the CDF. The CDF is calculated from the histogram of the image (Alarfaj et al., 2023). For a given intensity level *i*, the CDF *𝐶(𝑖)* is defined as in Equation 1:


(1) $$C\left(i\right)=\overset{i}{\underset{j=0}{\sum }}\frac{h\left(j\right)}{N}$$

where *h(j)* is the histogram count for intensity level *j*.

By expanding the brightness range, this technique enhances the visibility of elements within the image, proving especially beneficial for images characterized by low contrast resulting from illumination issues (Yin et al., 2022; Sun B. et al., 2024; Sun G. et al., 2024). The analysis of images benefits from the preprocessing step in the form of histogram equalization which increases the resolution and contrast in images and hence the accuracy of image analysis operations such as feature extraction, object detection and classification (Hanzla et al., 2024a; Hanzla et al., 2024b; Hanzla et al., 2024c). The transformation function maps the original intensity levels to the new equalized intensity levels. For an intensity level 𝑖, the new intensity level 𝑇(𝑖) is given by Equation 2:


(2) $$T\;\left(i\right)=\mathrm{round}\;\left(C\left(i\right)\times \left(L-1\right)\right)$$

**Figure 2:**
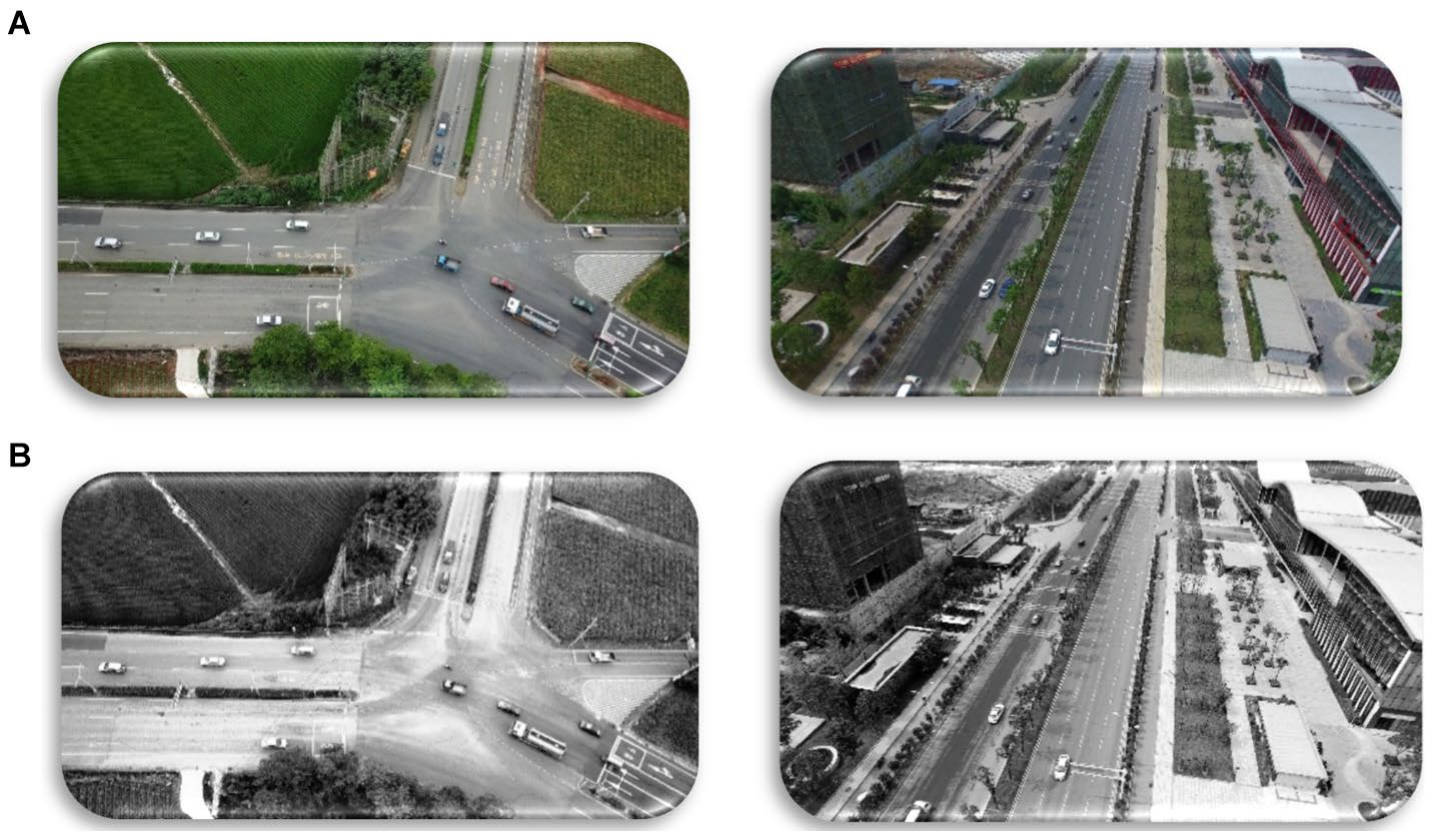
Histogram Equalization results over the VAID and UAVID datasets: **(A)** original Images; **(B)** Equalized images.

Where *C(i)* is the cumulative distribution function for intensity level *i. L* is the number of possible intensity levels in the image (typically 256 for an 8-bit image) (Figure 2).

### 3.3 Semantic segmentation

In many computer vision applications, including autonomous vehicles, medical imaging, virtual reality, and surveillance systems, image segmentation is essential. Images are divided into homogeneous sections using segmentation methods. Each area stands for a class or object. To improve item recognition on complex backgrounds, we compared two segmentation techniques.

#### 3.3.1 Contour-based segmentation

Contour-based segmentation emerged as a robust technique for partitioning images into meaningful regions by leveraging the detection and analysis of object boundaries (An et al., 2019; Khan et al., 2024a; Khan et al., 2024b). Through the application of edge detection algorithms and contour extraction methods, we successfully delineated objects within the images, enabling precise region-of-interest identification. First, the gradients of the image are computed to find areas with high spatial derivatives (Mi et al., 2023; Zhao et al., 2024; Chughtai and Jalal, 2023). The gradient magnitude 𝐺 and direction 𝜃 are calculated using the following Equations 3 and 4:


(3) $$G=\sqrt{G^{2}x+G^{2}}y$$

(4) $$Q=\arctan\;\left(\frac{\mathrm{Gy}}{Gx}\right)$$

Here, *Gx* and *Gy*, are the gradients which act along x and y axis, respectively. These gradients are normally estimated by convolution with the Sobel operators.

After that, non-maximum suppression is applied to thinning the edges to produce a binary edge map of a single pixel thick edges. This phase involves eliminating any gradient value that does not represent a peak in the search space. There are two thresholds, $T_{\mathrm{low}}$ and $T_{\mathrm{high}}$ which are used to classify edges as strong, weak and irrelevant. The classification is as follows using Equation 5 below:


(5) $$\left\{ \begin{array}{c} \mathrm{Strong}\;\mathrm{edge}\;\mathrm{if}\;G_{ij}\geq T_{\mathrm{high}}\\ \mathrm{weak}\;\mathrm{edge}\;\mathrm{if}\;T_{\mathrm{low}}\leq G_{ij}<T_{\mathrm{high}}\\ non\;\mathrm{relevant}\;\mathrm{if}\;G_{ij}<T_{\mathrm{low}} \end{array}\right.$$

**Figure 3:**
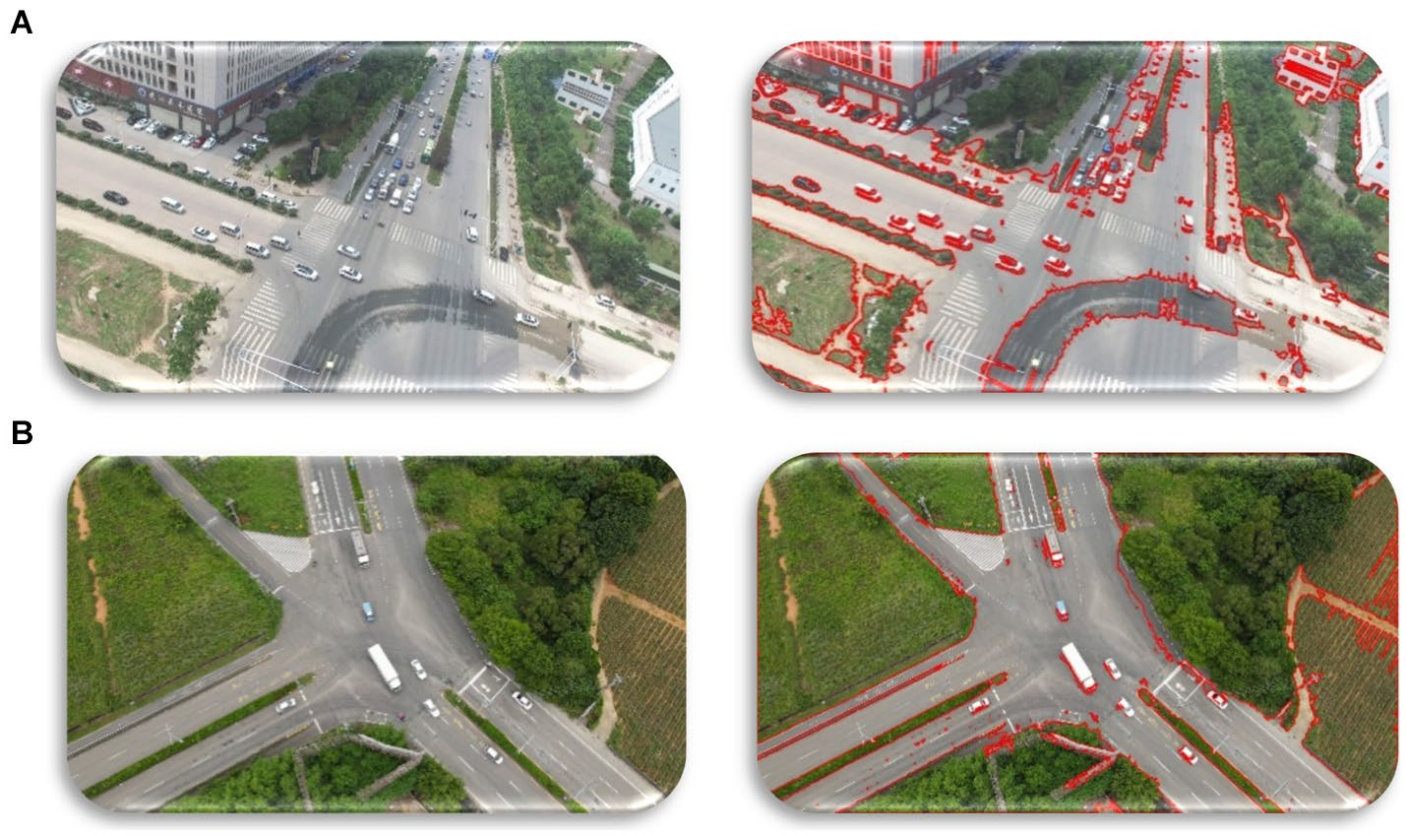
Contour-Based Segmentation used across UAVID and VAID datasets **(A)** original image **(B)** segmented image.

When evaluating the segmentation findings, this approach enabled precise segmentation free of omitted variability and other noise and fluctuation in light (Yang et al., 2022; Chen et al., 2022a; Chen et al., 2022b). By means of contour analysis techniques, the knowledge of spatial properties of objects was advanced to a substantial degree, thus improving segmentation results (Khan et al., 2024a; Khan et al., 2024b; Xu et al., 2022). Therefore, underlining its relevance in the improvement of the continued development of the most complicated systems of image analysis and interpretation, the efficacy of the contour-based segmentation as a technique employed in any domain, from object identification to medical imaging, indicates (Naseer et al., 2024) (Figure 3).

#### 3.3.2 Fuzzy C-mean segmentation

**Figure 4:**
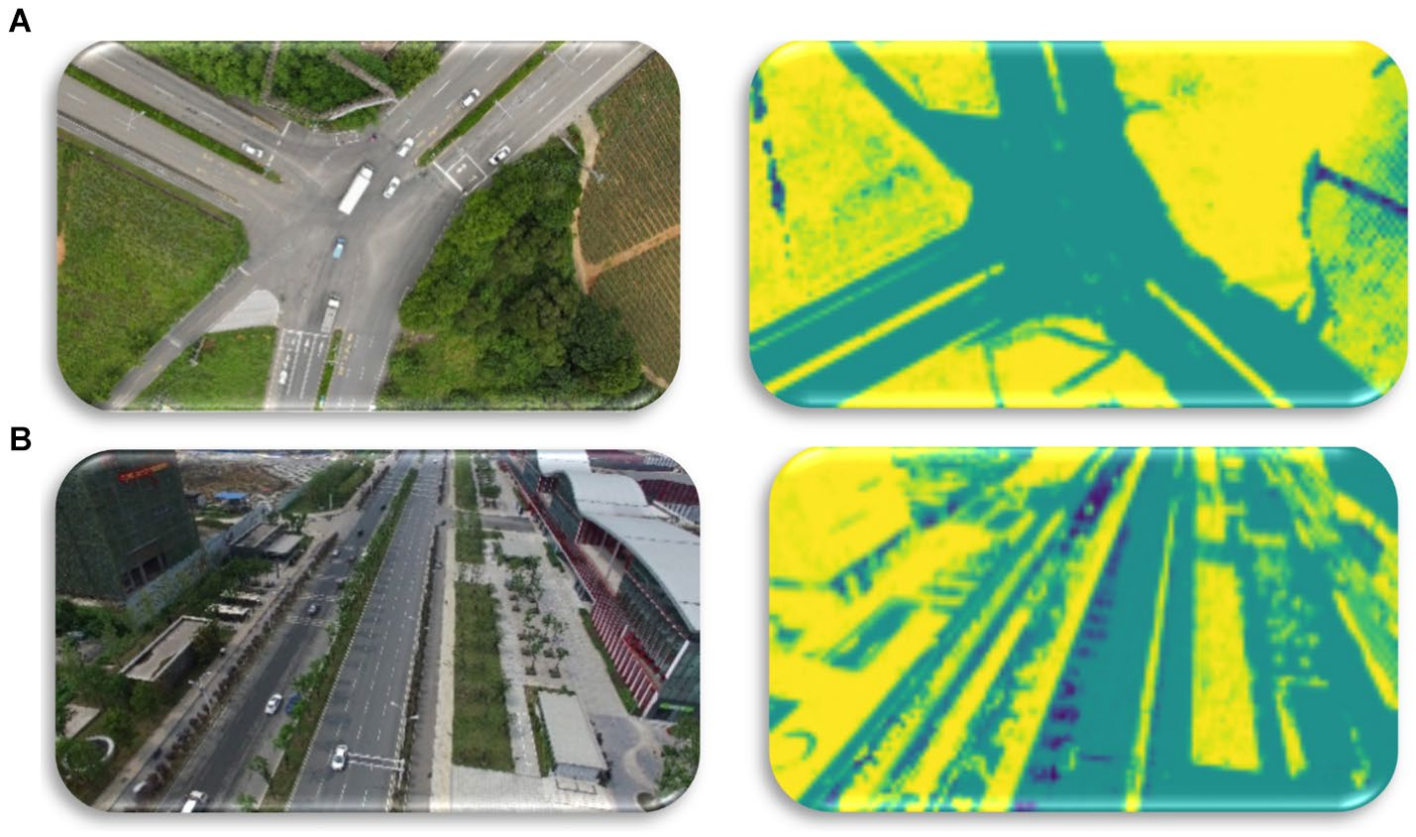
Using FCM for Semantic Segmentation over VAID and UAVID datasets **(A)** original image **(B)** segmented image.

To minimize the image’s complexity level, the image is segmented in this step using the Fuzzy C-Means (FCM) segmentation technique to separate the figure from the background (Zheng et al., 2024; Sun et al., 2019). Being the pixels belonging to multiple clusters fuzzy, FCM organizes the pixels in picture one or more clusters, (Saraçoğlu and Nemati, 2020; Sun et al., 2022). Membership degrees and clustering centers of the objectives are optimized through iterative amendments. As we have an existing, but limited set of elements, Q that can be divided into M clusters with the latter having known centers, the clustering method approach uses an adequate matrix, h, to express and measure the degree of belonging to each of the clusters (Xiao et al., 2024; Luo et al., 2022). Membership values h (j: y) (Javadi et al., 2018; Hashmi et al., 2024) show to what extent an element is committed to belonging to a certain cluster. The eq. Thus, where Kc is the number of fuzzy cluster components within the cluster, *L_f_*, the effectiveness index, is defined as follows in Equation 6:


(6) $$L_{f}=\Sigma_{a=1}^{M}\;\Sigma_{i=1}^{N}\;h_{ia}^{t}.dis_{ia}^{2}$$

where the equation depicts the quantification of the performance index concerning the membership matrix, distance, and cluster indices *a* and *i*. The proposed FCM segmentation result is shown in the Figure 4.

The CBS and FCM segmentation methods were evaluated in terms of computational cost and error rates determined using Equation 7.


(7) ErrorRate=1−accuracy

CBS outperforms FCM due to its excellent capability of dealing with datasets through various cluster shapes and different sizes (Sun et al., 2018; Xiao et al., 2023a; Xiao et al., 2023b). When it comes to contour-based segmentation methods, CBS is also effective to delineate the object boundaries and thus provides a more precise clustering compared to FCM (Al Mudawi et al., 2024). Therefore, while FCM solves the problem of uncertainty in the data point assignments by introducing membership degree of fuzziness, the CBS technique is sounder in offering good solution through contour analysis to perform accurate and adaptive clustering (Naseer and Jalal, 2024a,b). Additionally, CBS offers more flexibility in terms of change in the boundaries by way of parameterization where changes can be made more accurately to adequately respond to the nature of the data at hand (Zheng et al., 2023; Peng et al., 2023). Table 1 shows that CBS gets better results and more accurate for the picture segmentation on both VAID and UAVID datasets as compared to FCM method, which proves that CBS is efficient for the complicated image data. In terms of computational time and accuracy rates CBS is more efficient than FCM which can be useful in activities such as feature extraction as well as classification leading to better results in many operations.

**Table 1:** Error rate comparison of CBS and FCM.

Datasets	Error rate	
	FCM	CBS
VAID	0.32	0.22
UAVID	0.37	0.24

### 3.4 Vehicle detection via EfficientDet

For detecting vehicles, the EfficientDet, which is one of the most advanced object detection algorithms, was used to detect vehicles in aerial images. Therefore, EfficientDet maximizes the degree of accuracy and the related computing complexity by using the compound scaling technique. This covers consistently widening the depth, breadth, and backbone network resolution (Murthy et al., 2022; Sun et al., 2024). The effective feature fusion networks of many sizes augment the model architecture and help to improve the detection outcomes. Effective and quick integration of feature pyramid is achieved in EfficientDet by use of BiFPN (Bi-directional Feature Pyramid Network). This is particularly crucial when working with aerial images because things, such as automobiles, might vary substantially in scale (Naik Bukht et al., 2023). Using focus loss, a theory of correcting for class imbalance that emphasizes challenging cases helps the model’s performance to be better (Zhu, 2023; Bai et al., 2024). Consequently, in this study EfficientDet was trained using VAIDs and UAVIDs and acquired good accuracy and recall values for the vehicle identification task. The great stability of the model and its capacity to provide quick solutions for real-time applications in the realm of UAV based surveillance systems helped to demonstrate its further value (Mudawi et al., 2023).

**Figure 5:**
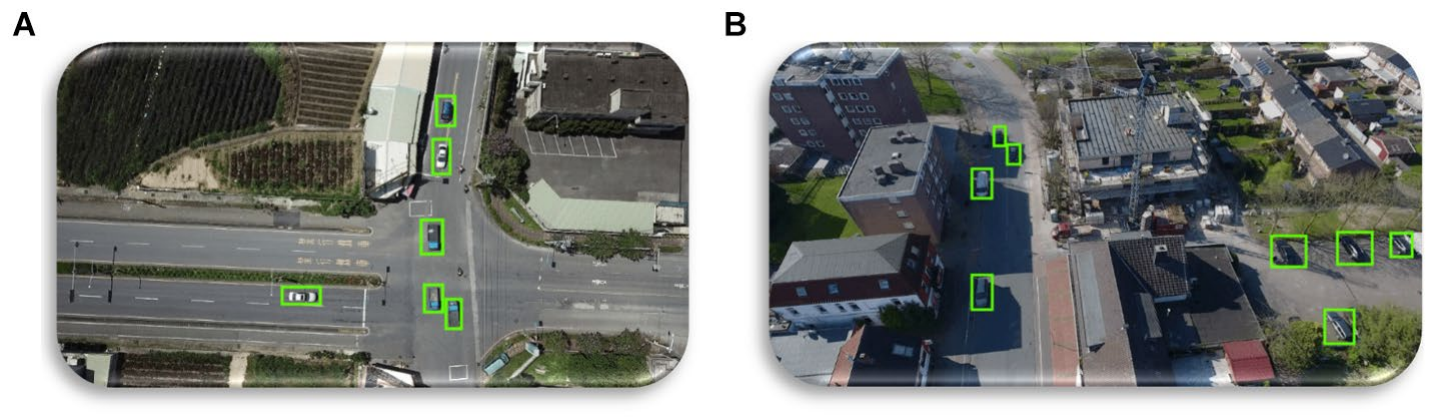
Vehicle Detection across **(A)** VAID and **(B)** UAVID datasets highlighted with green boxes using the EfficientDet method.

First, EfficientDet has a compound scaling method that helps to scale up not only the number of layers but also the width of the backbone network (Alshehri et al., 2024). This kind of approach enables the model to learn and understand the intricate hierarchical features and at the same time due to layer-wise learning rate factor computational complexity of the model does not increase greatly as the size of model increases (Naseer et al., 2024; Al Mudawi et al. 2024). In addition, EfficientDet incorporates efficient feature fusion schemes that help in the successful incorporation of the multi-scale information from different layers of the network architecture which in turn enhances the design to capture both the big picture and detail features. However, in EfficientDet, focus loss and scaling compound approaches have been used in the training process, which are effective in addressing the problems of class imbalance than in improving the model’s overall performances and calibration abilities. By such new architectural designs and training strategies, EfficientDet achieves the best correctness in object detection tasks while incurring less computational costs; hence, EfficientDet can be seen as an efficient and realistic solution (Mehla et al., 2024; Qiao et al., 2024) (Figure 5).

### 3.5 Feature extraction

This part describes feature extraction as a component, therefore clarifying the approaches proposed in this work. We used three strong feature extraction techniques: ORB, SIFT, AKAZE to extract various aerial image characteristics.

#### 3.5.1 AKAZE features

Renowned for speed and accuracy, AKAZE (Accelerated-KAZE) is a sophisticated feature extracting method. It works by spotting and characterizing local image properties unaffected by affine transformations, rotation, or scaling. AKAZE’s major value originates in the usage of nonlinear scale spaces for key point detection, which accelerates the process while keeping robustness. This makes it especially helpful in situations where computational efficiency is very crucial. In this study, AKAZE was employed to extract crucial points from the VAID and UAVID datasets, delivering a rich collection of features for vehicle recognition and classification tasks (Hanzla et al., 2024a; Hanzla et al., 2024b; Hanzla et al., 2024c).

**Figure 6:**
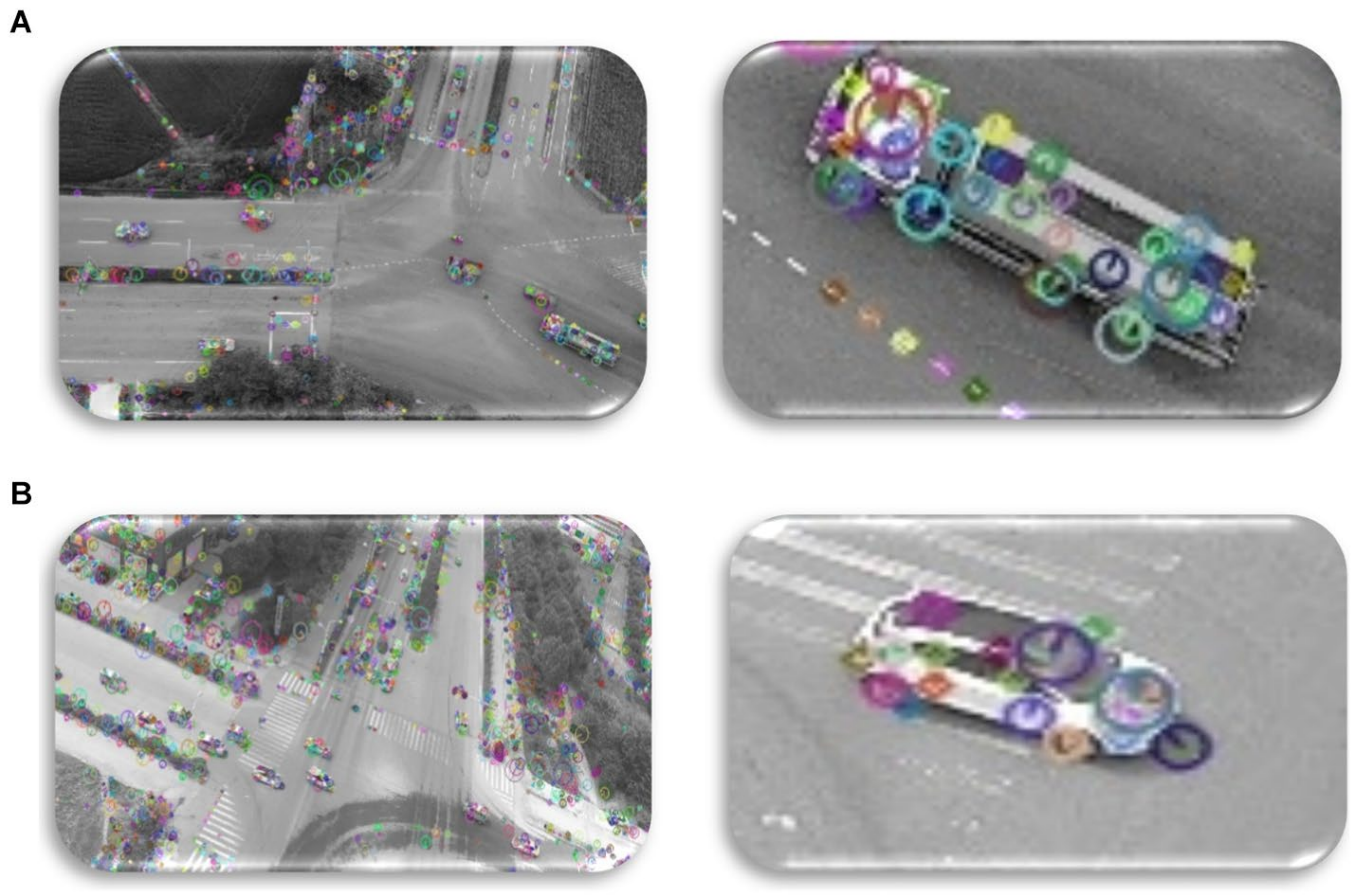
Using AKAZE for feature extraction across **(A)** VAID dataset and **(B)** UAVID dataset.

Sophisticated feature extraction techniques like AKAZE (Accelerated-KAZE) are often used in computer vision applications including registration, object identification, and image matching. AKAZE is resistant to changes in perspective and scene appearance because it finds and defines local image attributes that are invariant to scale, rotation, and affine transformations (Chughtai et al., 2024). Its acceleration via nonlinear scale space evolution and quick feature identification methods is one of its main advantages. This acceleration enables AKAZE to successfully produce high-dimensional feature descriptions while preserving accuracy and durability over diverse image sizes. Furthermore, AKAZE gives a large range of feature descriptors, including both local intensity information and spatial relationships among key places (Tareen and Saleem, 2018; Yin et al., 2024a; Yin et al., 2024b; Zhou et al., 2021). These characteristics are crucial for occupations requiring correct image matching and registration in applications ranging from augmented reality to panoramic stitching. The AKAZE feature extraction approach typically comprises critical procedures, including key point recognition, feature description, and matching, facilitating the finding of unique image features that allow dependable and accurate picture analysis (Ahmed and Jalal, 2024a; Ahmed and Jalal, 2024b). Figure 6 displays the AKAZE feature extraction process visually, demonstrating crucial steps in detecting and describing relevant spots across numerous sizes and orientations. Figure 6 presents instances of the obtained AKAZE characteristics, exhibiting their capacity to collect vital visual information and support powerful image analysis and interpretation (Chien et al., 2016; Yin et al., 2024a; Yin et al., 2024b).

#### 3.5.2 SIFT features

Feature Transform (SIFT). SIFT generates invariant to scale, rotation, and translation descriptors and detects significant areas in an image. SIFT is particularly helpful for evaluating aerial photos where vehicles might show different diameters and orientations, hence of this robustness (Kamal and Jalal, 2024). SIFT was used in this study on VAID and UAVID datasets to provide a collection of distinctive and consistent features that increase vehicle identification and classification accuracy (Mudawi et al., 2023).

**Figure 7:**
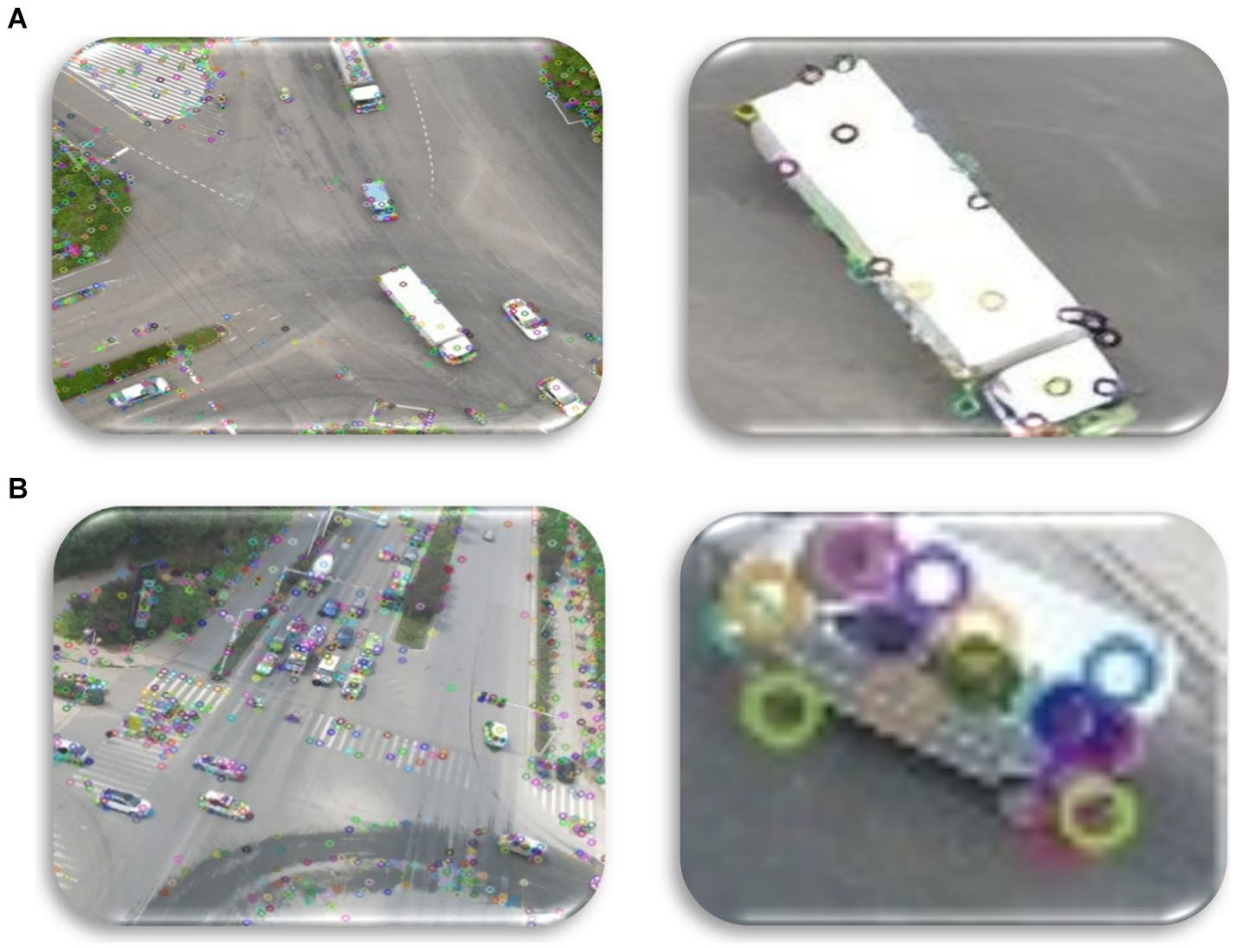
Using SIFT for feature extraction over **(A)** VAID dataset and **(B)** UAVID dataset.

Integration of a Scale Invariant Feature Transform (SIFT) method resulted in notable achievements. Therefore, it is feasible to find comparable patterns in other images by means of the reduction of the supplied image to a collection of points, which may be provided via SIFT. This approach performs well in obtaining such characteristics, even though specialized in scaling and rotation invariance (Ou et al., 2020; Chen et al., 2024; He et al., 2024). Figure 7 dissects the SIFT characteristics.

#### 3.5.3 ORB features

Another alike detector which is fast as SIFT is Oriented FAST and Rotated BRIEF (ORB). ORB uses the FAST keypoint detector and the following BRIEF descriptor with orientation compensation (Wang Z. et al., 2022). This combination makes it possible for ORB to carry out the tasks of feature detection as well as description in a very efficient and at the same time accurate manner suitable for use in vehicle detection systems that are mounted on UAVs. Presumably, ORB was employed for the extraction of features from the VAID and UAVID datasets, which in its turn constituted an overall robustness as well as the efficiency of the proposed method (Afsar et al. 2022; Abbas and Jalal, 2024).

Among all the corner and feature detectors, the Oriented FAST and Rotated BRIEF (ORB) extractor of features is quite robust in detecting critical areas. The primary detector used in the means is called the FAST (Features from Accelerated Segment Test). ORB renders rotational and dimensions consistency by interlinking the BRIEF description (Khan Tareen and Raza, 2023; Wang et al., 2023; Wu et al., 2023) at a closer level of detail. The following is the formula that the client needs to use to compute the patch moment or m_uv._ as described in Equation 8 below.


(8) $$m_{uv}=\underset{jk}{\sum }x^{u}y^{v}l\left(j,k\right)$$

Where $m_{uv}$ represents the moment of the image patch, *x* and *y* are the coordinates of the image pixels, *u* and *v* are the orders of the moments, and *l (j, k)* is the intensity of the pixel at position *(j, k)*. The center of the image is then computed using the following Equation 9:


(9) $$W=\frac{m_{10}}{m_{00}},\frac{m_{01}}{m_{00}}$$

Where W represents the coordinates of the image center, $m_{10}$ and $m_{01}$ are the first-order moments, and $m_{00}$ is the zeroth-order moment (the sum of all pixel intensities). Furthermore, the orientation (θ) is determined by the function as described in Equation 10:


(10) $$\theta =\mathrm{atan}\;\frac{m_{01}}{m_{00}}$$

**Figure 8:**
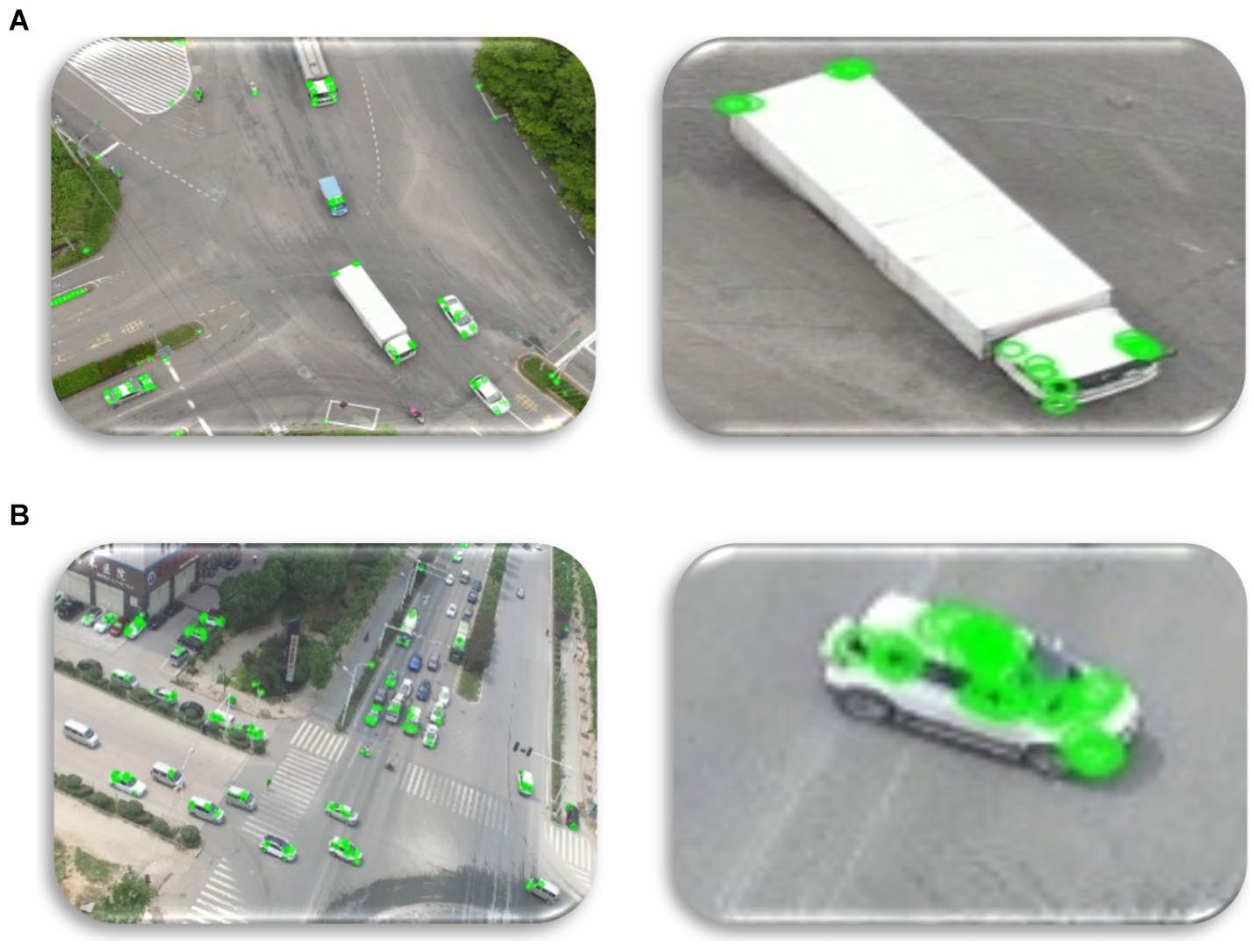
Using ORB for feature extraction over **(A)** VAID dataset and **(B)** UAVID dataset.

Where *θ* is the orientation angle, $m_{01}$ is the first-order moment along the y-axis, and $m_{10}$ is the first-order moment along the x-axis. The culmination of these computations results in the final extracted feature as depicted in Figure 8.

### 3.6 Vehicle classification

The proposed method reconciles the methods to identify and classify vehicles in aerial images acquired by unmanned aerial vehicles (Ali et al., 2022). Pre-processing the images using Contrast Limited Adaptive Histogram Equalization (CLAHE) and noise reduction methods first improves picture quality in the process. EfficientDet then is used to identify vehicles within the images. After that, feature extraction using ORB, SIFT, and AKAZE methods treats the spotted vehicles. Every found vehicle has a composite feature vector built from these properties (Cai et al., 2024; Abbasi and Jalal, 2024). Finally, using these feature vectors, a Convolutional Neural Network (CNN) classifier is trained to recognize the found vehicles. Following CNN-based classification, EfficientDet for detection and ORB, SIFT, and AKAZE for feature extraction presents great accuracy and robustness in vehicle detection and classification in UAV images (Jin et al., 2024; Hanzla et al., 2024a; Hanzla et al., 2024b; Hanzla et al., 2024c).

#### 3.6.1 Classification via CNN

EfficientDet was used to use Convolutional Neural Networks (CNNs) to improve detection performance post-identification in the vehicle classification stage. CNNs, which specialize in image recognition, shine at extracting discriminative features from found vehicles critical for complex classification. The design of CNN comprises convolutional, pooling, and fully connected layers that autonomously develop hierarchical representations, collecting crucial features for successful classification (Jahan et al., 2020; Hou S. et al., 2023; Hou X. et al., 2023).

During training, CNN was exposed to a labeled dataset, allowing it to generalize and make exact predictions on fresh vehicle photos. The end output of CNN recognized and classed cars, which is crucial for applications like traffic control and surveillance. Integrating CNNs into the proposed system considerably boosts its capabilities, creating doors for advanced applications in aerial vehicle surveillance. The CNN model’s design, training, and accuracy are thoroughly explained in succeeding parts, offering a clear grasp of its function in the study (Rajathi et al., 2022; Yusuf et al., 2024a; Yusuf et al., 2024b). The mathematical procedures involved in the convolutional layer are given by following Equation 11:


(11) $$z_{i,j}^{l}=f\;\left(\Sigma_{u=0}^{F-1}\;\Sigma_{v=0}^{F-1}\;x_{\left(i+u\right),\left(j+v\right)}^{\left(l-1\right)}.w_{u,v}^{\left(l\right)}+b^{l}\right)$$

where $z_{i,j}^{l}$ represents the activation at position *(i, j)* in the *l^th^* layer, *f* is the activation function, $x_{\left(i+u\right),\left(j+v\right)}^{\left(l-1\right)}$ is the input from the previous layer, $w_{u,v}^{\left(l\right)}$ is the weight, and $b^{l}$ is the bias. Similarly, the mathematical operations for the fully connected layer are expressed by the following Equation 12 below.


(12) $$a_{i}^{\left(l\right)}=f\;\left(\Sigma_{j=0}^{N\left(l-1\right)}.w_{i,j}^{\left(l\right)}\;x_{j}^{\left(l-1\right)}+b_{i}^{l}\right)$$

**Figure 9:**
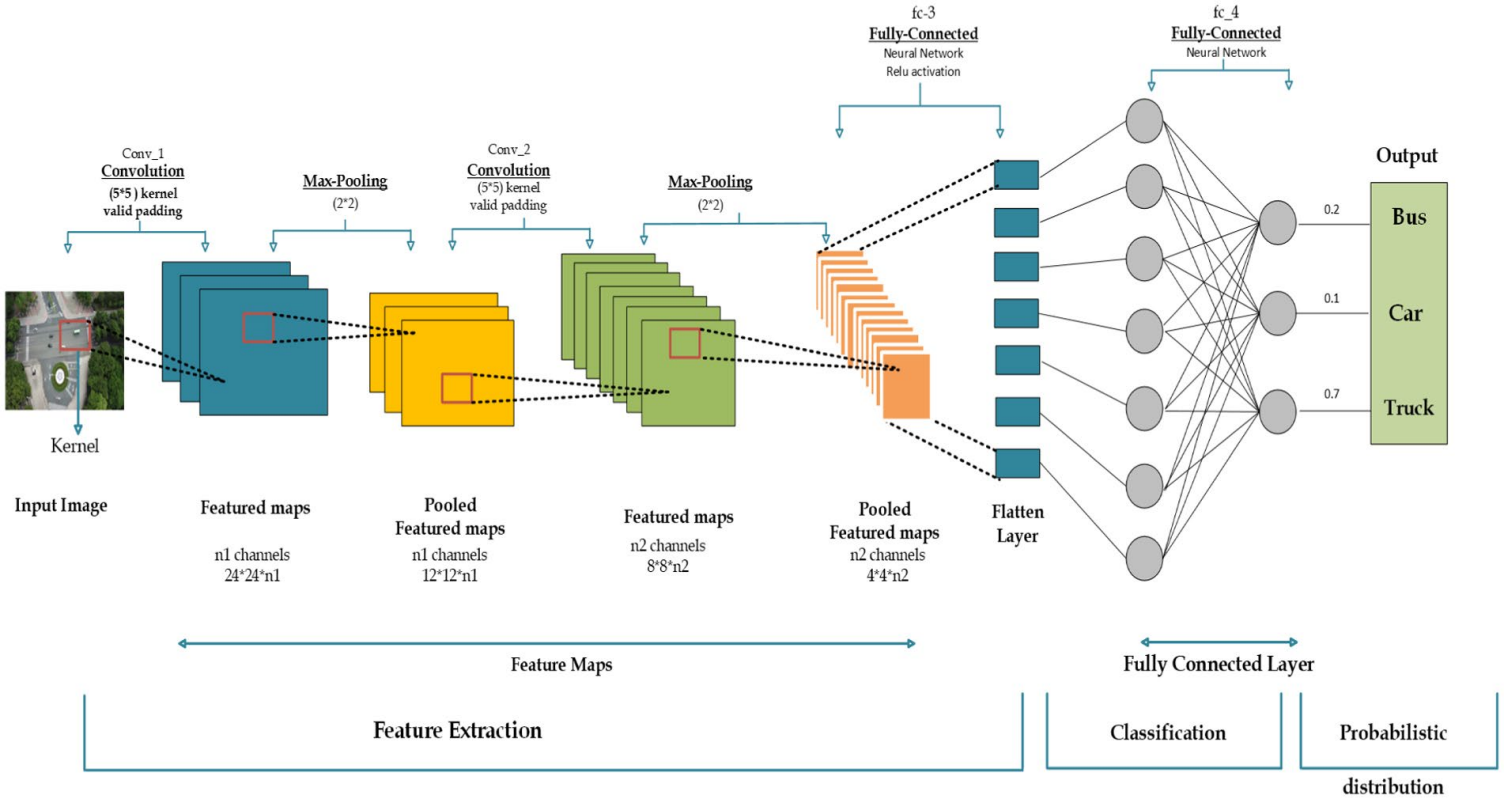
The architecture of CNN model for classification.

This equation represents the activation $a_{i}^{\left(l\right)}$ at position *i* in the *l^th^* fully connected layer, where *f* is the activation function, $w_{i,j}^{\left(l\right)}$ is the weight, $x_{j}^{\left(l-1\right)}$ is the input from the preceding layer, and $b_{i}^{l}$ is the bias (Figure 9). The detailed process is illustrated in Algorithm 1 that Harnessing the Strength of CNNs.

##### Algorithm 1 : Harnessing the Strength of CNNs.

**Input:** A = {i_1_, i_2_, …, i_n_}; img_frames **Output:** B = (n_0_, n_1_, …, n_N_): class_result. detected_vehicles ← []: Vehicle Detections feature_vectors ← []: Feat. Vector **Method:**video reader = Video Reader (‘video. mp4’) current frame = read(video_reader) **for** frameidx = 1 to size (current frame) reseeding = imresize (current frame[frame_idx], 768×768) segmented = FCM (reseeding) detected_vehicles ← EfficientDet (segmented) **for** vehidx = 1 to size(detected_vehicles) feat_vectors ← SIFT (detected_vehicles[vehidx]) feat_vectors ← ORB (detected vehicles[vehidx]) veh_classification = CNN (feat_vectors) **end forreturn** veh_classification end for **return** current frame

## 4 Experimental setup and datasets

### 4.1 Experimental setup

A PC running x64-based Windows 11 with an Intel Core i5-12500H 2.40GHz CPU, 24GB RAM, and other characteristics was used for the experiments. Spyder was employed to obtain the outcomes. The VEDAI and VAID datasets are three benchmark datasets that the system used to assess the performance of the suggested architecture. All three datasets undergo k-fold cross-validation to evaluate the dependability of our suggested system. In this section, the system is compared to other state-of-the-art technologies, the dataset is discussed, and the experiments are explained.

### 4.2 Dataset description

In the following subsection, we furnish comprehensive and detailed descriptions of each dataset utilized in our study. Each dataset is meticulously introduced, emphasizing its distinctive characteristics, data sources, and collection methods.

#### 4.2.1 UAVID dataset

**Figure 10:**
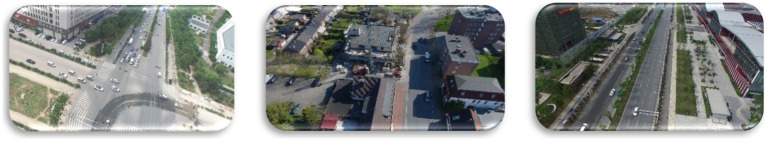
Sample images frame from the UAVID dataset.

The UAVID dataset offers a high-resolution view of urban environments for semantic segmentation tasks. It comprises 30 video sequences capturing 4 K images (meaning a resolution of 3,840 × 2,160 pixels) from slanted angles. Each frame is densely labeled with 8 object categories: buildings, roads, static cars, trees, low vegetation, humans, moving cars, and background clutter. The dataset provides 300 labeled images for training and validation, with the remaining video frames serving as the unlabeled test set. This allows researchers to train their models on diverse urban scenes and evaluate their performance on unseen data (Yang et al., 2021) (Figure 10).

#### 4.2.2 VAID dataset

**Figure 11:**
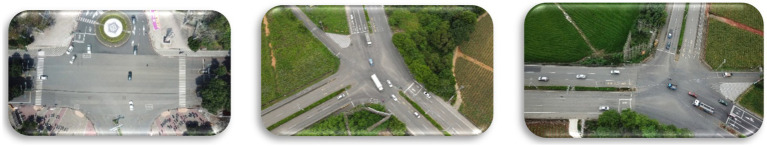
Sample images frame from the VAID dataset.

The VAID collection featured six separate vehicle image categories such as minibus, truck, sedan, bus, van, and car (Lin et al., 2020). These images are obtained by a drone in different illumination circumstances. The drone was situated between 90 and 95 meters above the earth’s surface. The resolution of images taken at 23.98 frames per second is 2,720 × 1,530. The dataset offers statistics on the state of the roads and traffic at 10 sites in southern Taiwan. The traffic images illustrate an urban setting, an educational campus, and a suburban town (Figure 11).

## 5 Results and analysis

### 5.1 Experiment I: semantic segmentation accuracy

The CBS and FCM algorithms were compared and assessed in terms of segmentation accuracy and computational time. FCM requires training on a bespoke dataset, increasing the model’s computing cost as compared to CBS. Furthermore, CBS produced superior segmentation results than FCM, therefore we utilized the CBS findings for future investigation. Table 2 shows the accuracy of both segmentation strategies.

**Table 2:** Accuracies comparison of FCM and CBS segmentation.

Datasets	Segmentation Accuracies	
	FCM	CBS
VAID	0.69	0.85
UAVID	0.67	0.80

### 5.2 Experiment II: precision, recall, and F1 scores

The effectiveness of vehicle detection and tracking has been assessed using these evaluation metrics, namely *Precision, Recall, and F1 score* as calculated by using Equations 13, 14, and 15 below:


(13) $$\mathrm{Precision}=\frac{\sum TP\;}{\sum TP+\sum FP}$$

(14) $$\mathrm{Recal}l=\frac{TP\;}{TP+FN}$$

(15) $$F1\;\mathrm{Score}=\frac{2\left(\mathrm{Precision}\times \mathrm{Recall}\right)}{\left(\mathrm{Precision}+\mathrm{Recall}\right)}$$

Table 3 shows vehicle detection’s precision, recall, and F1 scores. True Positive indicates how many cars are effectively identified. False Positives signify other detections besides cars, whereas False Negatives shows missing vehicles count. The findings indicate that this suggested system can accurately detect cars of varying sizes (Table 4).

**Table 3:** Overall accuracy, precision, recall, and F1-score for vehicle detection over the UAVID dataset.

Vehicle class	Precision	Recall	F1-score
Pickup	0.987	0.977	0.985
Aircraft	0.993	0.989	0.975
Vans	0.943	0.961	0.956
Car	0.912	0.914	0.909
Truck	0.945	0.980	0.955
Others	0.965	0.971	0.968
Mean	**0.956**	**0.964**	**0.958**

**Table 4:** Overall accuracy, precision, recall, and F1-score for vehicle detection over the VAID dataset.

Vehicle class	Precision	Recall	F1-score
Sedan	0.965	0.979	0.975
Minibus	0.988	0.972	0.950
Truck	0.979	0.991	0.981
Bus	0.952	0.975	0.962
Trailer	0.981	0.966	0.946
Car	0.956	0.909	0.927
Mean	**0.970**	**0.965**	**0.965**

### 5.3 Experiment IV: confusion matrix

Tables 5, 6 provide comprehensive confusion matrices that illustrate the performance of our vehicle classification methods on the UAVID and VAID datasets, respectively. These matrices reveal the precision of our classification by indicating how frequently vehicles from different classes are correctly identified (diagonal elements) as opposed to being misclassified (off-diagonal elements). Table 5 highlights that our proposed method achieved high precision across various vehicle classes, culminating in an impressive overall mean precision of 0.966. Similarly, Table 6 showcases the accuracy of our suggested method, achieving a mean precision of 0.97. This demonstrates robust performance across multiple vehicle types. These results underscore the efficacy of our classification algorithms in accurately identifying and categorizing different vehicle classes, thus affirming their reliability and effectiveness in diverse applications.

**Table 5:** Confusion matrix illustrating the precision of our proposed vehicle classification approach on the UAVID dataset.

Vehicle class	Pickup	Tractor	Vans	Car	Truck	Others
Pickup	0.97	0	0	0	0	0
Aircraft	0.02	0.98	0	0	0	0
Vans	0	0.01	0.96	0.02	0	0
Car	0	0	0.04	0.92	0	0.01
Truck	0	0.03	0	0	0.97	0
Others	0	0	0	0	0	0.98
**Mean: 0.966**						

**Table 6:** Confusion matrix demonstrated our suggested vehicle categorization method’s accuracy using the VAID dataset.

Vehicle class	Sedan	Minibus	Truck	Bus	Vans	Car
Sedan	0.98	0.01	0.01	0	0	0
Minibus	0	0.94	0.02	0.03	0	0
Truck	0	0.01	0.98	0	0	0
Bus	0.01	0.02	0	0.98	0	0
Trailer	0.01	0	0	0	0.99	0
Car	0.03	0.01	0.01	0	0	0.95
**Mean: 0.97**						

### 5.4 Experiment V: ablation study experiment

The ablation study in Table 7 evaluates the performance of our model by systematically removing individual components. Each row represents a version of the model with a specific component removed, and the corresponding accuracy is measured on the UAVID and VAID datasets. This table demonstrates the importance of each component in achieving high accuracy.

**Table 7:** Ablation study experiment of all methods on UAVID and VAID datasets.

Experiment	Histogram equalization	FCM	AKAZE	ORB	SIFT	EfficientDet	CNN (ResNet)	UAVID	VAID
Full model (Baseline)	Yes	Yes	Yes	Yes	Yes	Yes	Yes	96.6	97
Without histogram equalization	No	Yes	Yes	Yes	Yes	Yes	Yes	95	94.2
Without FCM	Yes	No	Yes	Yes	Yes	Yes	Yes	92	91.5
Without AKAZE	Yes	Yes	No	Yes	Yes	Yes	Yes	93	92.3
Without ORB	Yes	Yes	Yes	No	Yes	Yes	Yes	94	93.1
Without SIFT	Yes	Yes	Yes	Yes	No	Yes	Yes	91	90.8
Without EfficentDet	Yes	Yes	Yes	Yes	Yes	No	Yes	88	87.6
Simple CNN instead of ResNet	Yes	Yes	Yes	Yes	Yes	Yes	No	90	89.5
Without traditional features (CNN)	Yes	Yes	No	No	No	Yes	Yes	85	84.2
Without traditional features (Simple)	Yes	Yes	No	No	No	Yes	No	80	79.8

The ablation study presented in Table 7 demonstrates the robustness and effectiveness of the proposed model components on the UAVID and VAID datasets. Removing individual components such as histogram equalization, FCM, AKAZE, ORB, SIFT, and EfficientDet significantly degrades the model performance, indicating their essential contributions. Notably, the absence of EfficientDet results in the most substantial drop in accuracy, underscoring its critical role in the detection pipeline. Additionally, substituting the ResNet backbone with a simpler CNN architecture leads to a noticeable decline in performance, highlighting the importance of using a sophisticated feature extractor. These findings validate the necessity of the integrated components and their synergistic effect in achieving high accuracy for UAV-based vehicle detection.

### 5.5 Comparison with other state-of-the-art methods

Table 8 compares our proposed model’s performance with existing state-of-the-art methods. The figures for our model are consistent with those in Table 7.

**Table 8:** Comparison of the proposed method with existing methods on UAVID and VAID datasets.

Methods	UAVID	VAID
Mandal et al. (2019)	53.95	–
du Terrail et al. (2018)	82.52	–
Wang et al. (2020)	89.21	–
Lin et al. (2020)	–	88.1
Hou et al. (2023)	75.54	–
**Our proposed model**	**96.6**	**97**

Research welcoming cross-validation for the results portrays the robustness of the model for the vehicle detection and aerial images classification. Application of EfficientDet, which is well-known for object identification of various sizes and appearances intensity, gives our approach more credibility. Furthermore, obtaining important features of the surrounding environment along with the form and texture of the objects improves categorization accuracy to the maximum extent.

### 5.6 Detailed analysis of the comparison with other state-of-the-art methods

Table 8 provides a comparison of our proposed method with existing methods on the UAVID and VAID datasets. The results highlight the significant improvement in performance achieved by our approach:

1. Superior Performance on UAVID Dataset: Our suggested model obtains an accuracy of 96.6%, which is substantially greater than the accuracies produced by existing state-of-the-art approaches such as Mandal et al. (53.95%), Terrail et al. (82.52%), Wang et al. (89.21%), and Hou et al. (75.54%). This highlights the stability and efficacy of our approach in managing the intricacies of the UAVID dataset.
2. Outstanding Results on VAID Dataset: For the VAID dataset, our technique obtains an accuracy of 97%, exceeding Lin et al.’s method, which achieved 88.1%. This suggests that our technique is extremely successful in vehicle identification and classification under varied environmental circumstances and vehicle kinds as documented in the VAID dataset.

The benefit of our suggested strategy is further underlined utilizing EfficientDet for vehicle detection. EfficientDet’s compound scaling method, efficient feature fusion, and usage of focus loss contribute to its outstanding performance in object identification tasks, as seen by the high accuracy and recall rates attained on both datasets. Moreover, the combination of modern methods such as Histogram Equalization, FCM, AKAZE, ORB, and SIFT in our model further strengthens its power to effectively recognize and categorize automobiles in aerial images.

Overall, the results in Table 8 clearly demonstrate the superiority of our proposed method over existing methods, providing a robust solution for vehicle detection and classification in UAV-based surveillance systems.

## 6 Discussion/research limitation

**Figure 12:**
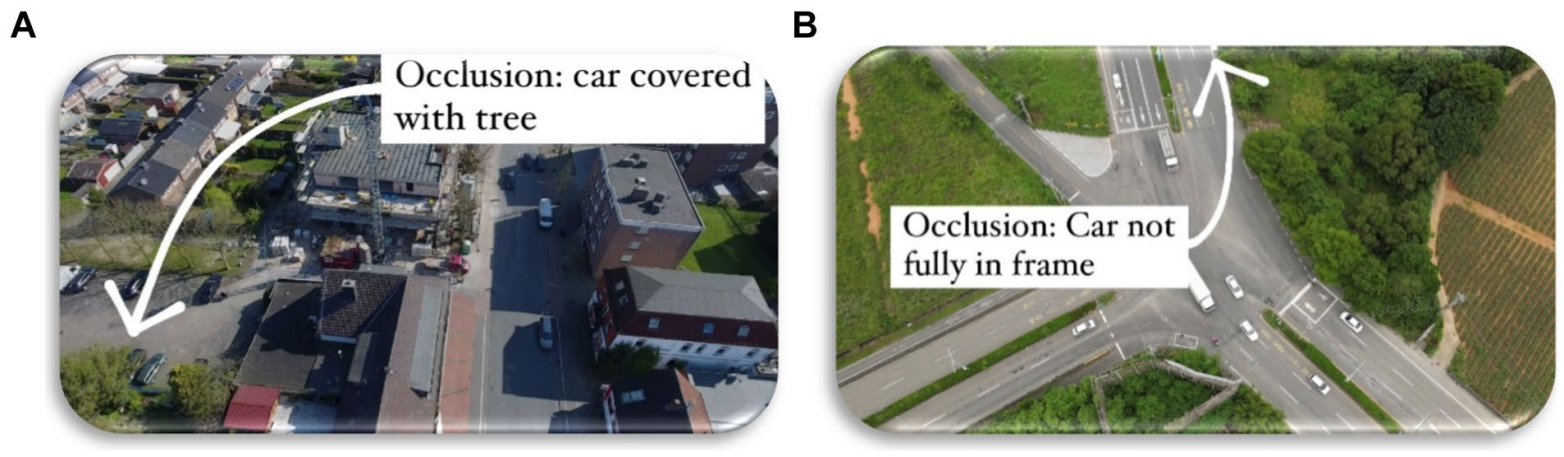
Limitations of our proposed model: **(A)** vehicle not detected due to Occlusion; car covered with tree **(B)** Car not fully in frame.

For effective traffic monitoring based on aerial images, our suggested model is an efficient solution. While catering to high-definition aerial images, object detection is one of the most difficult problems. To get efficient results, we devised a technique that combines contour based semantic segmentation with CNN classification. However, the suggested technique has significant limitations. First and foremost, the system has only been evaluated with RGB shots acquired during the daytime. Analyzing video or pictorial datasets in low-light conditions or at night can further confirm this proposed technique as a lot of researchers already have succeeded with such datasets. Furthermore, our segmentation and identification system have problems with partial or complete occlusions, tree-covered roadways, and similar items (Figure 12).

## 7 Conclusion

This study presents a novel method for classifying and identifying vehicles in aerial image sequences by utilizing cutting-edge approaches at each stage. The model starts by applying Histogram Equalization and noise reduction techniques to pre-process aerial images. After segmenting the image using Fuzzy C-Means (FCM) and Contour based segmentation (CBS) to reduce image complexity, EfficientDet is used for vehicle detection. Oriented FAST, Rotated BRIEF, Scale Invariant Feature Transform (SIFT), and AKAZE (Accelerated-KAZE) are used to extract features from detected vehicles (ORB). Convolutional Neural Networks (CNNs) are used in the classification phase to create a strong system that can correctly classify cars. Promising results are obtained with the proposed methodology: 96.6% accuracy on the UAVID dataset and 97% accuracy on the VAID dataset. Future enhancements to the system could involve incorporating additional features to boost classification accuracy and conducting training with a broader range of vehicle types. Moving forward, our aim is to explore reliable methodologies and integrate more features into the system to enhance its efficacy, aspiring for it to become the industry standard across a spectrum of traffic scenarios.

## Data Availability

Publicly available datasets were analyzed in this study. This data can be found here: https://www.kaggle.com/datasets/dasmehdixtr/uavid-v1.
